# The “Aging Effect” of BMI on Cardiorespiratory Fitness: A New Insight on Functional Evaluation in Obesity

**DOI:** 10.3390/jcm12227183

**Published:** 2023-11-20

**Authors:** Francesca Battista, Daniel Neunhaeuserer, Anna Centanini, Andrea Gasperetti, Giulia Quinto, Marco Vecchiato, Elia Bianchi, Anna Chiara Frigo, Silvia Bettini, Roberto Vettor, Luca Busetto, Andrea Ermolao

**Affiliations:** 1 Department of Medicine, Sports and Exercise Medicine Division, University of Padova, 35128 Padova, Italy; francesca.battista@unipd.it (F.B.); anna.centanini@aulss8.veneto.it (A.C.); andrea.gasperetti@aopd.veneto.it (A.G.); giulia.quinto@phd.unipd.it (G.Q.); marco.vecchiato.4@phd.unipd.it (M.V.); bnclei@unife.it (E.B.); andrea.ermolao@unipd.it (A.E.); 2 Center for the Study and the Integrated Treatment of Obesity, Padova Hospital, 35128 Padova, Italy; silvia.bettini@aopd.veneto.it (S.B.); roberto.vettor@unipd.it (R.V.); luca.busetto@unipd.it (L.B.); 3 Clinical Network of Sport and Exercise Medicine of the Veneto Region, 35128 Padova, Italy; 4 Department of Cardiac, Thoracic and Vascular Sciences, University of Padova, 35128 Padova, Italy; annachiara.frigo@unipd.it; 5 Department of Medicine, Internal Medicine 3, Padova University Hospital, 35128 Padova, Italy

**Keywords:** cardiopulmonary exercise test, aerobic capacity, VO_2_ peak, maximal oxygen consumption

## Abstract

Cardiorespiratory fitness (CRF) is a strong predictor of morbidity and mortality in patients with obesity. This study investigates the CRF range and its clinical determinants in patients with obesity. Moreover, a practical proposal for CRF interpretation is provided. In this study, 542 patients (69% females) with BMI ≥ 30 kg/m^2^ performed an incremental cardiopulmonary exercise test (CPET). Patients had a median (IQR) age of 47.0 (6.2) years with a mean BMI of 41.7 ± 6.7 kg/m^2^. Normal values curves of VO_2_peak/kg showed a median (IQR) of 20.3 (37.6) mL/min/kg. The lower-quartile threshold of VO_2_peak/kg was at 17.9 mL/min/kg. Analysis of covariance revealed that VO_2_peak/kg inversely correlates with age and BMI with a significant age × BMI interaction effect (all *p* < 0.0001); as BMI class increases, CRF decreases, but a smaller age-related decline in VO_2_peak/kg is observed. A multivariate logistic regression demonstrated that belonging to the lower quartile of VO_2_peak/kg was independently determined by age (OR 2.549, 95% CI 1.205–5.392, *p* < 0.0001) and BMI (OR 5.864, 95% CI 2.920–11.778, *p* < 0.0001) but not by comorbidities. At very high BMI, the effect of age on functional capacity is lower, suggesting that BMI acts as an “aging factor” on CRF. Age and BMI, but not comorbidities, are independent determinants of low VO_2_peak/kg.

## 1. Introduction

Cardiorespiratory fitness (CRF) is a solid marker of cardiovascular and general health, as well as a strong predictor of cardiovascular and all-cause mortality both in the general population and in patients with chronic diseases [1]. Cardiopulmonary exercise test (CPET) is the gold standard for the evaluation of CRF and the VO_2_peak results from the integrated efficiency of the systems involved in oxygen delivery and utilization [2], as well as the subjects’ working capacity. Low CRF is associated with a higher risk of mortality, and increasing CRF has a positive impact on lowering this risk [3,4,5]. The linkage between CRF and mortality has also been described in patients with obesity in whom higher CRF improved overall health regardless of body weight [6]. Systematic measurement of functional capacity in clinical practice is, therefore, essential for the evaluation of individuals with chronic diseases.

Obesity is a globally spreading disease that strongly affects the ability to carry out daily life activities and limits the quality of life, both with regard to basic and complex activities [7]. These limitations depend on several factors such as altered biomechanics and posture, balance impairment, reduced muscle strength, and poor work capacity due to low CRF [8]. Therefore, people living with obesity are exposed to a high risk of disability and, as recently described, VO_2_peak included in the standard Edmonton Obesity Staging System can improve the power of this clinical classification in stratifying the severity of obesity [7]. The assessment of CRF in clinical settings is particularly encouraged due to the high predictive power of cardiovascular disease and mortality in patients with obesity [3]. It is known, indeed, that the high risk linked to fatness may be partially counteracted by fitness and that maintaining or improving CRF is associated with a significant reduction in cardiovascular risk [9]. Obesity is characterized by an accelerated aging process as evidenced by the fact that even young individuals, including children, living with obesity, can have chronic diseases that usefully develop in adulthood or during aging, i.e., diabetes mellitus type 2, high blood pressure, and cancer [10]. These observations are also supported by numerous similarities between the pathophysiological mechanisms of obesity and those related to aging, such as inflammation, the oxidoreductive state, autophagy, and mitochondrial dysfunction [11]. Indeed, both aging and obesity negatively affect functional capacity [7,12]. The negative impact of obesity on all components of the oxygen transport system (i.e., muscular, cardiovascular, pulmonary, metabolic, and autonomic) involved in the response to exercise leads to a lower CRF [13]. CRF is associated with disability and cardio-metabolic syndrome [1], and obesity is itself associated with several comorbidities that may affect functional capacity and life expectancy and can in turn be improved by the practice of regular physical exercise [6,14]. Moreover, it is demonstrated that CRF is a stronger predictor of mortality and cardiovascular outcomes than traditional risk factors, such as obesity, arterial hypertension, type 2 diabetes, and dyslipidemia [1], and even more notably, it has been described that the greatest risk reduction is achieved by moving from the lowest fitness level to the next [15]. It is known, indeed, that men with obesity who are at least moderately fit have a very low risk of death, compared to normal-weight men who are unfit [16]. Furthermore, it has been described that in people with obesity, CRF, but not fat mass, is associated with functional limitations and that unfit subjects with low CRF had a greater impact of disability on activities of daily living [17,18]. Nevertheless, to date, the real distribution and normal range of CRF assessed by maximal cardiopulmonary exercise testing are still unknown for the population with obesity, as well as the respective impact of age, sex, and comorbidities on this important parameter. Furthermore, although it is known that people living with obesity have a reduced CRF with a higher absolute aerobic power, the quantification of this reduction has never been reported in a large population of subjects with various degrees of obesity and assessed with the gold standard method for functional capacity evaluation. Moreover, current formulas for the CRF classification seem to misrepresent the real functional impairment of patients with obesity, especially for the most severe forms. This lack of knowledge currently limits clinical evaluations and decision-making and calls for specific research investigating aerobic capacity in obesity and its determinants for this population.

The aim of this study is therefore to describe the distribution of VO_2_peak assessed by cardiopulmonary exercise testing (CPET) among subjects with a large range of obesity, according to sex, age, and BMI classes. In addition, the impact of comorbidities on CRF will be explored.

## 2. Materials and Methods

This is an observational cross-sectional study involving a large sample of patients with obesity consecutively referred to the Sports and Exercise Medicine Division of the University of Padova for functional evaluation. Study participants were enrolled between February 2014 and February 2018. The study included patients with different degrees of obesity, all showing a body mass index (BMI) > 30 kg/m^2^. Exclusion criteria were conditions contraindicating the CPET execution [19]. A large number of the samples met the selection criteria, which may provide an indication for bariatric surgery. Anamnestic data about family history, presence of cardiovascular risk factors, diabetes mellitus, obstructive sleep apnea (OSA), arthropathy, depression, asthma, and dyslipidemia were collected. The current pharmacological therapy was also reported. Patients’ physical activity was evaluated by questions commonly used in clinical settings and related to the type of exercise, training frequency, and workout intensity. The initial examination included anthropometric measurements, and all subjects performed maximal CPET. The whole population was divided into BMI classes, based on the WHO obesity classification (1st, 2nd, 3rd obesity degrees and patients with BMI > 45 kg/m^2^). The CPET was fully explained to each patient, with a clarification of risks and benefits, before all subjects gave written informed consent in accordance with the Declaration of Helsinki. The protocol was approved by the “Padova Ethical Committee for Clinical Research” (2892P, 10 June 2013).

### 2.1. CPET Protocol

CPET was mainly carried out on a treadmill or, alternatively, in a small number of cases, on a cycle-ergometer (generally when patients presented musculoskeletal problems that prevented the achievement of an adequate intensity level on the treadmill test). Before each test, the system was calibrated according to the indications of the manufacturer, and each patient performed a resting spirometry. The ventilatory and gas exchange parameters were sampled breath by breath (Jaeger-Masterscreen-CPX exhaled gas analysis system, Carefusion) at rest, during the whole exercise phase, and four minutes of recovery. An incremental ramp exercise protocol was used, specifically adapted from the “Bruce protocol”, i.e., a standard Bruce ramp incremental protocol performed after a 5 min constant-load phase (at 2.7 km/h and 0% slope). In the case of patients performing a cycle-ergometer test, a standardized ramp protocol was used (+15 Watts/min). The following were considered as criteria of exhaustion, defining a maximal exercise test: a respiratory exchange ratio (RER) ≥ 1.10, Borg rating of perceived exertion 18/20, and a reached heart rate ≥ 85% of the predicted value by age [20]. All patients who did not reach at least one criterion were excluded from the study. The subjects were continuously monitored by a 12-lead electrocardiogram during the exercise and the recovery phases. Blood pressure was measured by auscultatory method at rest, during exercise, and during the recovery phase. Peripheral oxygen saturation was assessed by pulse oximeter in each test phase. Functional capacity was reported as the absolute aerobic power (VO_2_peak L/min), the VO_2_peak in relation to body weight (VO_2_peak mL/kg/min), as well as the percentage of the predicted value for age, sex, and weight (VO_2_peak%). VO_2_peak% was calculated by dividing the obtained VO_2_peak value by the expected one according to Wasserman’s equation [21]. Other reported CPET parameters are oxygen uptake efficiency slope (OUES), VE/VCO_2_ slope, oxygen pulse (VO_2_/HR mL/min/bpm), and breathing reserve (expressed as the difference between calculated maximal voluntary ventilation and measured maximal ventilation at peak exercise, expressed as percentage).

### 2.2. Statistical Analyses

All data were collected in an EXCEL spreadsheet and analyzed by a statistician with the SAS 9.4 program (SAS Institute Inc., Cary, NC, USA) for Windows. The statistical significance level was set at 5%. Data related to RER, VE/VCO_2_ slope, breathing reserve, VO_2_peak%, VO_2_/HR, VO_2_peak/kg, VO_2_peak, and OUES were compared with BMI categories by variance analysis, as they all showed normal distribution. A covariance analysis model (ANCOVA) was performed to evaluate the BMI category effect and the impact of sex and age on cardiorespiratory CPET indices, including individual factors and their interactions. In the case of statistical significance, coupling comparisons were made using Bonferroni’s method (Tukey). Potential predictors of VO_2_/kg < 17.8 mL/min/kg (lower-quartile cut-off value of data distribution) were assessed by a univariate binary logistic regression model. The statistically significant predictors have been incorporated into a multivariable binary logistic regression model. The results are presented with a statistical significance “*p*” value, odds ratio estimation, and 95% confidence interval.

## 3. Results

### 3.1. Population Characteristics

Clinical characteristics of the population are summarized in Table 1.

The studied sample involved 542 patients with obesity (69% females) with a mean age (±SD) of 45.5 ± 12.3 years. The mean BMI was 41.7 ± 6.7 kg/m^2^. CPET was mostly performed on a treadmill (84%). The most frequent comorbidity was arterial hypertension, which affects about one-third of the studied population. Arthropathy, dyslipidemia, and type 2 diabetes were present in approximately 20% of the population. OSA and hypothyroidism showed a prevalence of 14% in the whole population. All hypothyroid patients were euthyroid with hormonal supplementation. Asthma and depression affected 8.3% and 6.6% of patients, respectively. The chi-squared test revealed a higher prevalence of arterial hypertension (48.8% vs. 27.1%), type 2 diabetes (27.1% vs. 14.9%), dyslipidemia (27.7% vs. 16.0%), and OSA (31.3% vs. 6.4%), in man than in women, while hypothyroidism was more frequent in women than in men (17.6% vs. 6.6%). For the remaining comorbidities, the sex difference was not statistically significant.

### 3.2. Functional Capacity: Distribution and Determinants

**Figure 1:**
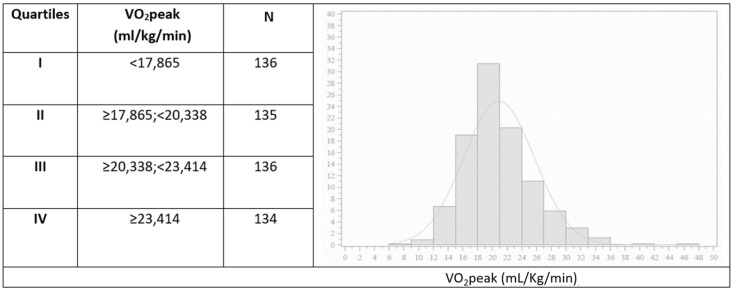
VO_2_peak/kg (mL/kg/min) distribution in the whole population. The figure shows the distribution of the VO_2_peak/kg in the whole population of patients with obesity. Discrete VO_2_peak/kg values derived from the quartile cut-off point may serve as a reference for the comparison and evaluation of functional capacity in patients with obesity.

Evaluating patients’ aerobic capacity, VO_2_peak/kg in the whole population was on average 20.9 ± 4.8 mL/kg/min, while the absolute VO_2_peak was 2.41 ± 0.6 L/min. The VO_2_peak value represented 101.3 ± 16.7% of predicted for age and sex. As shown in Figure 1, VO_2_peak/kg was found normally distributed in this population. The subdivision into quartiles allowed us to identify 17.865 mL/kg/min as the upper value of the lower quartile.

Functional parameters were then described according to BMI classes, and results are displayed in Table 2. The VO_2_peak% was significantly lower for the BMI > 45 kg/m^2^ category when compared to those in the 30–35 kg/m^2^ (*p* < 0.017) and 40–45 kg/m^2^ group (*p* < 0.001). The difference between VO_2_peak/kg values was highly significant among all pairwise comparisons (*p* < 0.0001), excluding that between the 35–40 kg/m^2^ and 40–45 kg/m^2^ groups.

**Figure 2:**
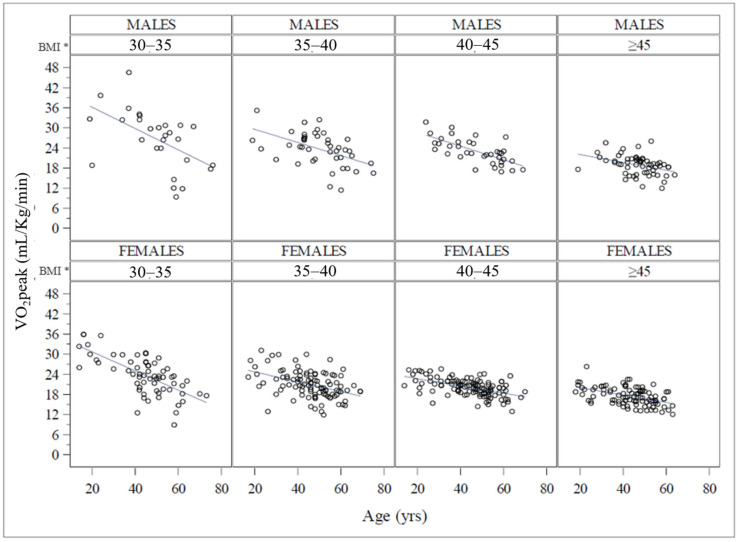
Analysis of covariance (ANCOVA) for VO_2_peak/kg among BMI classes corrected for age and sex. The figure shows the relationship between age and functional capacity expressed as VO_2_peak/kg for each sex and in different BMI groups. It clearly shows the change in this relationship among BMI classes and also in different sexes. The figure visually shows a kind of “aging effect” determined by BMI. Indeed, it is appreciable that young subjects with very high BMI have a similar functional capacity to older subjects also suffering from obesity, but with a lower BMI. * BMI is expressed as kg/m^2^.

The absolute VO_2_peak was significantly higher in the group of patients with BMI > 45 kg/m^2^ when compared with those of BMI 30–35 kg/m^2^ (*p* = 0.004) and 35–40 kg/m^2^ (*p* = 0.002). The covariance analysis (ANCOVA) illustrated in Figure 2 showed that the VO_2_peak/kg decreased with increasing age and BMI.

**Figure 3:**
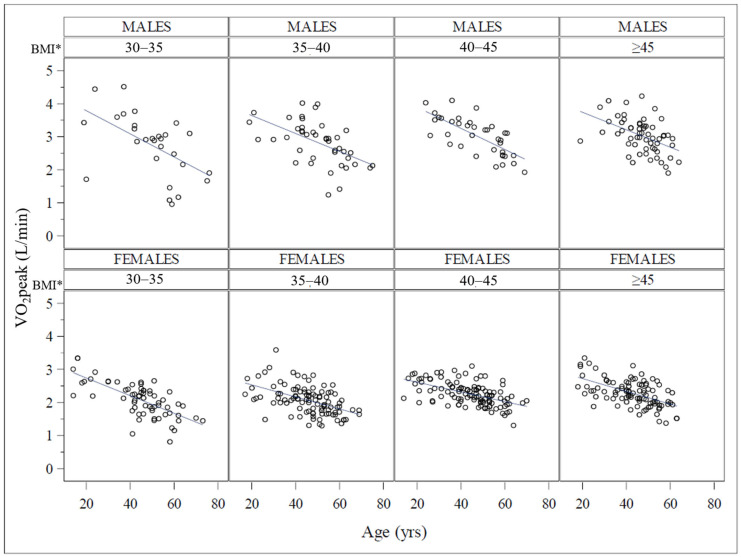
Analysis of covariance (ANCOVA) for VO_2_peak among BMI classes corrected for age and sex. The figure shows the relationship between age and absolute aerobic capacity expressed as VO_2_peak (L/min) for each sex and in different BMI groups. It clearly shows the different slope of this relationship between age sexes. * BMI is expressed as kg/m^2^.

The effect of these two variables is observable in both sexes, with an impact of age in all categories but less evident in patients with higher BMI. In fact, the only significant interaction term was found between age and BMI (*p* < 0.0001). The same analysis conducted for the absolute VO_2_peak (Figure 3) showed that the aerobic power increases as the BMI increases with a higher maximum oxygen consumption in the upper BMI group. Conversely, the absolute VO_2_peak decreased as age increased, with similar trends in the different BMI classes, but with a steeper slope in males than in females. In this case, the only significant interaction term was between age and sex (*p* < 0.001). Females showed significantly lower VO_2_peak than males for each BMI class.

Univariate logistic regression analysis showed that the odds of being in the lower VO_2_peak/kg quartile were significantly determined by age, BMI > 45 kg/m^2^, and the presence of more than two comorbidities (Table 3).

A multivariate logistic regression confirmed the independent role of age and BMI in determining functional capacity but excluded the independent relationship with comorbidities (Table 4). In particular, the <39 years class, compared to the two oldest population groups (i.e., 47–54 and >54 years classes), showed an odds ratio and the respective confidence interval of approximately 2.549 (1.205–5.392) and 6.132 (2.859–13.153), respectively. Regarding the BMI classes, the comparison between the 30–35 kg/m^2^ category and the >45 kg/m^2^ category was statistically significant, with an odds ratio and the related 95% confidence interval of 5.864 (2.920–11.778).

### 3.3. Cardiovascular and Cardiopulmonary Efficiency

Cardiovascular and cardiopulmonary efficiency parameters (Table 2) showed significant differences among BMI classes. The oxygen pulse was progressively lower passing from the lowest to the highest BMI class. The OUES was different for all pairwise comparisons, excluding that between the 30–35 kg/m^2^ and 35–40 kg/m^2^ BMI groups. The VE/VCO_2_slope differed significantly only between the lowest and highest BMI classes (*p* = 0.022), while breathing reserve varied significantly among all groups and decreased progressively with increasing BMI.

## 4. Discussion

The main findings of this study are that patients with mild, moderate, and severe obesity have a reduced functional capacity. The average VO_2_peak/kg is 20.9 mL/kg/min and by subdividing the population into quartiles of weight-related CRF, a VO_2_peak/kg equal to 17.9 mL/kg/min is identified as the upper limit of the first quartile and may represent a reference point for the definition of “reduced functional capacity” in patients with obesity. Furthermore, our data suggest that, despite a normal reduction in functional capacity with aging, the higher the BMI values, the smaller the effect of age on VO_2_peak/kg, while the impact of BMI is higher for younger people, explaining most part of the reduction in functional capacity. Thus, BMI seems to act as an early functional “aging factor”.

When compared with the reference population (provided by the FRIEND study), the functional capacity measured in this population shows an average value well below the reference value in the same age group, both in men and women [12]. Several studies have corroborated the evidence that CRF is one of the major prognostic predictors, even when compared to the main cardiometabolic risk factors [1]. Therefore, it is crucial to identify subjects with reduced CRF and to implement actions to remove causes of functional limitation and to improve functional capacity. In particular, it has been described that in patients with obesity, absolute aerobic power increases with increasing BMI, while the maximal aerobic capacity relative to body weight (VO_2_peak/kg) is significantly reduced. Conversely, an inverse effect is detectable after bariatric surgery, when absolute aerobic power slightly decreases and functional capacity significantly increases [22]. Nevertheless, VO_2_peak/kg is the parameter that really reflects the ability to carry out daily activities and represents the strongest long-term prognostic marker for both disability and mortality [1]. In the present study, we have described clinical aspects associated with the variability of functional capacity among patients with obesity. Similar to people with normal weight, VO_2_peak/kg decreases with increasing age and BMI in both men and women with obesity. However, the higher the BMI values, the smaller the effect of age on VO_2_peak/kg. As shown in Figure 2, the decrease as a function of age is smaller for the higher BMI class, and conversely, the impact of BMI is higher for younger people. This seems to suggest that BMI acts as an early functional aging factor that later in life is exceeded by the age itself. This pathophysiological pattern has been recently outlined by Tam et al. who described obesity and aging as “two sides of the same coin” [11]. This review of literature, indeed, pointed out that obesity is a disease that accelerates aging, and this is established in genetic and epigenetic modifications, mitochondrial function, redox homeostasis, immunity, body composition, inflammation, and also functional decline [11]. Our study highlights that the CRF of patients with severe obesity is markedly reduced in young individuals. The hypothesis that obesity leads to early aging is supported by clear evidence regarding the increased risk of age-related diseases, such as arterial hypertension and type 2 diabetes, even in children living with obesity [23]. In addition, in this population, the declining trend of VO_2_peak/kg with increasing age and BMI was similar between the sexes, and sex was not associated with the likelihood of belonging to the lowest quartile of VO_2_peak/kg. Nevertheless, females showed, as expected, a lower CRF than males for each BMI class, indicating a more severe functional limitation. It is known that a lower VO_2_peak/kg can lead to a lower functional ability and barriers affecting the quality of life, especially when the values result close to or lower than those required to carry out activities of daily living. Former findings described that the age-related drop in VO_2_peak/kg follows a non-linear trend with a steeper decrease after 45 years, and this behavior is similar in men and women [24]. On the other hand, absolute VO_2_peak decreases as age increases with a steeper slope in men than in women; our study confirms this sex effect on absolute VO_2_peak [25]. In addition, the increasing BMI values are correlated to the decrease in VO_2_peak/kg regardless of age and sex. Nevertheless, in a multivariate logistic regression model, only the highest BMI class was a significant and independent determinant of the lowest VO_2_peak/kg quartile, indicating that extremely low values of functional capacity are more frequent among people with higher BMI. Furthermore, it should be noted that metabolic, cardiovascular, and cardiopulmonary efficiency parameters also vary significantly as BMI increases (Table 2), evidencing the numerous obesity-related cardiorespiratory changes. These results are in line with previous literature, but they clearly and originally show the significant difference in these parameters among different BMI classes. In particular, there is a progressive decrease in RER at peak exercise, suggesting the decreased ability to select substrates during exercise (metabolic inflexibility) [26]. The progressive decrease in VE/VCO_2_slope may be associated with the increase in CO_2_ during exercise, while the reduction in breathing reserve may stay in line with the mechanical ventilatory constraint already described in these subjects [27]. Finally, the oxygen pulse at peak exercise is also progressively reduced with increasing BMI; this is currently of uncertain clinical meaning but may also indicate possible cardiac involvement and/or cardiovascular inefficiency in patients with severe obesity [22].

For all these reasons and for many other concomitant factors, it is reasonable to trust in findings indicating that obesity reduces life expectancy and increases disability and limitations during activities of daily living [28,29]. Previous knowledge has widely highlighted that obesity is a complex condition that triggers neuro-endocrine, inflammatory, and metabolic mechanisms that inexorably lead to the development of cardiovascular diseases [30,31]. The role of physical activity in improving health and preventing disability has been largely described [32,33], and recently, it has been summarized that physical exercise improves the cardio-metabolic profile, general health, and CRF in people with obesity [34]. However, the actual interaction between comorbidities and CRF has not yet been sufficiently explained. In this regard, despite the univariate logistic regression analysis (Table 3) showed a correlation between the presence of a high number of comorbidities and a VO_2_peak value of less than 17.86 mL/kg/min, the multivariable logistic regression analysis (Table 4) does not provide statistically significant values. Therefore, those who present a high number of comorbidities (≥2) are more frequently in the lowest quartile of VO_2_peak/kg, but this effect is more attributable to age and high BMI values, which are the strongest and independent determinants of CRF.

### Limitations and Perspectives

Despite the large population, it was not possible to identify precise reference values due to the narrow age range and asymmetric age distribution in different BMI classes. Furthermore, longitudinal data were not available at the moment, limiting the possibility of identifying a precise CRF level linked to a higher risk of cardiovascular events or all-cause mortality. Therefore, this is a preliminary analysis aimed at investigating the main functional parameters detectable in the cardiopulmonary exercise test specific for patients with obesity. This work can serve as a guide for further studies characterized by a greater sample heterogeneity, in order to establish reference values of functional and aerobic capacity that could be effectively implemented in the functional evaluation of this population. Future studies may also consider the role of physical activity in determining CRF and hard clinical endpoints. The spread of the practice of clinicians to collect the level of physical activity as a *vital sign* will allow to have a large number of data and to better define the role of this parameter in patients’ health. Indeed, recent recommendations on exercise prescription endorsed by the European Society for the Study of Obesity pointed out that any type of exercise may improve functional capacity in patients with overweight or obesity [34]. A further limitation is that the majority of patients performed the test before undergoing bariatric surgery: They were therefore selected according to eligibility criteria such as BMI > 40 kg/m^2^ or greater than 35 kg/m^2^ if associated with comorbidities. To avoid this selection bias, statistical analyses were adjusted.

## 5. Conclusions

In conclusion, the present study quantifies the functional impairment of people with varying degrees of obesity, by using the gold standard method for the evaluation of cardiorespiratory fitness and efficiency, i.e., CPET. These data suggest that age and BMI, more than comorbidities, are the main determinants of CRF. In particular, the highest levels of BMI appear a stronger modifier than age in affecting functional capacity, and this is also evident in young individuals. A possible BMI-related “aging effect” could thus be hypothesized, independently from the presence of main comorbidities linked to obesity. This study indirectly highlights the important role of functional evaluation and the associated therapeutic exercise prescription in these patients. To improve the management of these patients, functional aspects must also be considered and implemented in order to guarantee a higher life expectancy and a better quality of life.

## Figures and Tables

**Table 1:** Clinical characteristics of the whole population.

Age, Years	45.5 ± 12.3
BMI, kg/m^2^	41.7 ± 6.7
Hypertension, N (%)	183 (33.8)
Arthropathy, N (%)	111 (20.5)
Dyslipidemia, N (%)	106 (19.6)
Type 2 diabetes, N (%)	101 (18.6)
Hypothyroidism, N (%)	76 (14.0)
OSAS, N (%)	76 (14.0)
Asthma, N (%)	45 (8.3)
Depression, N (%)	36 (6.6)
CPET	
VO_2_peak/kg (mL/min/kg)	20.9 ± 4.8
VO_2_ peak (L/min)	2.41 ± 0.6
VO_2_peak%	101.3 ± 16.7
RER	1.15 ± 0.09
OUESs (mL/LogL)	2424.4 ± 651.5
VE/VCO_2_ slope	26.3 ± 3.5

Data are presented as mean ± SD. Abbreviations: BMI = body mass index; VO_2_ peak = absolute volume of oxygen consumption at peak; VO_2_peak/kg = weight-related volume of oxygen consumption at peak; VO_2_peak% = percentage of oxygen consumption at peak compared to the predicted value for age and gender (Wasserman’s equation); RER = respiratory exchange ratio; OUESs = oxygen uptake efficiency slope; VE/VCO_2_ slope = VE/carbon dioxide production (VCO_2_) slope.

**Table 2:** Functional parameters described according to BMI classes.

BMI (kg/m^2^)	30–35		35–40		40–45		>45	ANOVA
N	92		144		151		155	*p*
VO_2_peak (L/min)	2.28 ± 0.74	*	2.29 ± 0.61	*	2.45 ± 0.52		2.56 ± 0.57	0.0002
VO_2_peak (mL/min/kg)	24.58 ± 6.85	*§‡	21.76 ± 4.48	*	20.83 ± 3.13	*	18.0 ± 2.81	<0.0001
VO_2_peak%	103 ± 0.21;	*	101 ± 0.18		105 ± 0.14	*	97 ± 0.13	0.007
OUES (ml/LogL)	2109.6 ± 658.1	*§	2285.3 ± 634.1	*§	2479.9 ± 551.2	*	2686.3 ± 645.3	<0.0001
RER	1.15 ± 0.08		1.16 ± 0.11	*	1.15 ± 0.12		1.13 ± 0.08	0.037
VO_2_/HR%	107.70 ± 27.55	*§	100.38 ± 22.13	*§	99.20 ± 19.58	*	85.02 ± 20.83	<0.0001
VE/VCO_2_ slope	27.16 ± 3.93	*	26.17 ± 3.87		26.39 ± 4.22		25.82 ± 3.16	0.031
BR	28.67 ± 17.65	*§‡	27.00 ± 15.10	*§	21.39 ± 13.22		21.74 ± 13.58	<0.0001

Data are presented as mean ±SD. Abbreviations: BMI = body mass index; VO_2_ peak = absolute volume of oxygen consumption at peak; VO_2_peak/kg = weight-related volume of oxygen consumption at peak; VO_2_peak% = percentage of oxygen consumption at peak compared to the predicted value for age and gender (Wasserman’s equation); OUES = oxygen uptake efficiency slope; RER = respiratory exchange ratio; VO_2_/HR% = oxygen pulse (%of the predicted for age and sex); VE/VCO_2_ slope = VE/carbon dioxide production (VCO_2_) slope; BR = breathing reserve. The significance of post-hoc analyses (Bonferroni test *p* < 0.05) is expressed as symbols: ‡ 35–40; § 40–45; * >45.

**Table 3:** Determinants of low VO_2_peak/kg by a univariate logistic regression model.

		VO_2_peak/kg (mL/kg/min)			
		<17.86 (Q1)	>17.86	OR (95% CI)	*p*
Gender	Males	34 20.5%	132 79.5%	1.445 (0.931–2.244)	0.1011
	Females	102 27.1%	274 72.9%		
Age (years)	<39	15 11.6%	114 88.4%	-	
<39 vs.	39–47	29 20.7%	111 79.3%	1.985 (1.010–3.903)	<0.0001
	47–54	33 26.0%	94 74.0%	2.668 (1.367–5.206)	
	>54	59 40.4%	87 59.6%	5.153 (2.740–9.693)	
BMI (kg/m^2^)	30–35	14 15.2%	78 84.8%	-	
30–35 vs.	35–40	27 18.6%	117 81.4%	1.286 (0.635–2.605)	<0.0001
	40–45	24 15.9%	127 84.1%	1.053 (0.514–2.156)	
	>45	71 45.8%	84 54.2%	4.709 (2.457–9.027)	
Number of comorbidities	0	28 16.0%	147 84.0%	-	
0 vs.	1	37 24.7%	113 75.3%	1.719 (0.993–2.976)	0.0006
	2	33 27.6%	86 72.3%	2.015 (1.140–3.561)	
	>2	38 38.8%	60 61.2%	3.325 (1.875–5.898)	

The univariate regression model reported in this table shows the correlation between sex, age, BMI, comorbidities, and the odds of being in the lower functional capacity quartile. Abbreviations: BMI = body mass index (kg/m^2^); VO_2_peak/kg = weight-related volume of oxygen consumption at peak.

**Table 4:** Determinants of the lower VO_2_peak/kg quartile by a multivariate logistic regression model.

		OR (95% CI)	*p*
Age (years) <39 vs.	39–47	1.885 (0.909–3.906)	<0.0001
	47–54	2.549 (1.205–5.392)	
	>54	6.132 (2.859–13.153)	
BMI (kg/m^2^) <35 vs.	35–40	1.154 (0.551–2.418)	<0.0001
	40–45	1.064 (0.502–2.255)	
	>45	5.864 (2.920–11.778)	
Number of comorbidities 0 vs.	1	1.401 (0.762–2.579)	0.5447
	2	1.148 (0.592–2.229)	
	>2	1.562 (0.774–3.150)	

The multivariate regression model reported in this table shows the independent determinants of the odds of being in the lower functional capacity quartile. Abbreviations: BMI = body mass index (kg/m^2^).

## Data Availability

Data are available upon reasonable request from the corresponding author in order to match with privacy policy.

## References

[1] Ross R., Blair S.N., Arena R., Church T.S., Després J.P., Franklin B.A., Haskell W.L., Kaminsky L.A., Levine B.D., Lavie C.J. (2016). Importance of Assessing Cardiorespiratory Fitness in Clinical Practice: A Case for Fitness as a Clinical Vital Sign: A Scientific Statement from the American Heart Association. Circulation.

[2] Gibbons R.J., Balady G.J., Beasley J.W., Bricker J.T., Duvernoy W.F., Froelicher V.F., Mark D.B., Marwick T.H., McCallister B.D., Thompson P.D. (1997). ACC/AHA Guidelines for Exercise Testing: A report of the American College of Cardiology/American Heart Association task force on practice guidelines (Committee on Exercise Testing). J. Am. Coll. Cardiol..

[3] Lee D.C., Artero E.G., Sui X., Blair S.N. (2010). Mortality trends in the general population: The importance of cardiorespiratory fitness. J. Psychopharmacol..

[4] Blair S.N. (1989). Physical fitness and all-cause mortality. A prospective study of healthy men and women. JAMA.

[5] Laukkanen J.A., Kurl S., Salonen R., Rauramaa R., Salonen J.T. (2004). The predictive value of cardiorespiratory fitness for cardiovascular events in men with various risk profiles: A prospective population-based cohort study. Eur. Heart J..

[6] Fogelholm M. (2010). Physical activity, fitness and fatness: Relations to mortality, morbidity and disease risk factors. A systematic review. Obes. Rev..

[7] Bettini S., Quinto G., Neunhaeuserer D., Battista F., Belligoli A., Milan G., Gasperetti A., Vettor R., Ermolao A., Busetto L. (2021). Edmonton Obesity Staging System: An improvement by cardiopulmonary exercise testing. Int. J. Obes..

[8] Capodaglio P., Castelnuovo G., Brunani A., Vismara L., Villa V., Capodaglio E.M. (2010). Functional limitations and occupational issues in obesity: A review. Int. J. Occup. Saf. Ergon..

[9] Ortega F.B., Cadenas-Sanchez C., Lee D., Ruiz J.R., Blair S.N., Sui X. (2018). Fitness and Fatness as Health Markers through the Lifespan: An Overview of Current Knowledge. Prog. Prev. Med..

[10] Hruby A., Hu F.B. (2015). The Epidemiology of Obesity: A Big Picture. Pharmacoeconomics.

[11] Tam B.T., Morais J.A., Santosa S. (2020). Obesity and ageing: Two sides of the same coin. Obes. Rev..

[12] Kaminsky L.A., Arena R., Myers J. (2015). Reference Standards for Cardiorespiratory Fitness Measured With Cardiopulmonary Exercise Testing. Mayo Clin. Proc..

[13] Arena R., Cahalin L.P. (2014). Evaluation of cardiorespiratory fitness and respiratory muscle function in the obese population. Prog. Cardiovasc. Dis..

[14] Conway B., Rene A. (2004). Obesity as a disease: No lightweight matter. Obes. Rev..

[15] Kodama S., Saito K., Tanaka S., Maki M., Yachi Y., Asumi M., Sugawara A., Totsuka K., Shimano H., Ohashi Y. (2009). Cardiorespiratory Fitness as a Quantitative Predictor of All-Cause Mortality and Cardiovascular Events in Helathy Men and Women: A Meta-Analysis. JAMA.

[16] Blair S.N. (2009). Physical inactivity: The biggest public health problem of the 21st century. Br. J. Sports Med..

[17] Bouchard D.R., McGuire K.A., Davidson L., Ross R. (2011). Cardiorespiratory fitness, obesity, and functional limitation in older adults. J. Aging Phys. Act..

[18] O’Neill D., Forman D.E. (2020). The importance of physical function as a clinical outcome: Assessment and enhancement. Clin. Cardiol..

[19] Fletcher G.F., Ades P.A., Kligfield P., Arena R., Balady G.J., Bittner V.A., Coke L.A., Fleg J.L., Forman D.E., Gerber T.C. (2013). Exercise standards for testing and training: A scientific statement from the American heart association. Circulation.

[20] Guazzi M., Adams V., Conraads V., Halle M., Mezzani A., Vanhees L., Arena R., Fletcher G.F., Forman D.E., Kitzman D.W. (2012). Clinical Recommendations for Cardiopulmonary Exercise Testing Data Assessment in Specific Patient Populations. Circulation.

[21] Wasserman K., Hansen J.E., Sue D.Y., Stringer W.W., Sietsema K.E., Sun X.-G., Whipp B.J. (2011). Priciples of Exercise Testing and Interpretation: Including Pathophysiology and Clinical Applications.

[22] Neunhaeuserer D., Gasperetti A., Savalla F., Gobbo S., Bullo V., Bergamin M., Foletto M., Vettor R., Zaccaria M., Ermolao A. (2017). Functional Evaluation in Obese Patients Before and After Sleeve Gastrectomy. Obes. Surg..

[23] Gurnani M., Birken C., Hamilton J. (2015). Childhood Obesity: Causes, Consequences, and Management. Pediatr. Clin. N. Am..

[24] Jackson A.S., Sui X., O’Connor D.P., Church T.S., Lee D.C., Artero E.G., Blair S.N. (2012). Longitudinal cardiorespiratory fitness algorithms for clinical settings. Am. J. Prev. Med..

[25] Stathokostas L., Jacob-Johnson S., Petrella R.J., Paterson D.H. (2004). Longitudinal changes in aerobic power in older men and women. J. Appl. Physiol..

[26] Battista F., Belligoli A., Neunhaeuserer D., Gasperetti A., Bettini S., Compagnin C., Marchese R., Quinto G., Bergamin M., Vettor R. (2021). Metabolic Response to Submaximal and Maximal Exercise in People with Severe Obesity, Prediabetes, and Diabetes. Obes. Facts.

[27] Borasio N., Vecchiato M., Quinto G., Battista F., Neunhaeuserer D., Ermolao A. (2022). Correspondence regarding ‘Ventilatory efficiency in athletes, asthma and obesity’: Different ventilatory phenotypes during exercise in obesity?. Eur. Respir. Rev..

[28] Peeters A., Barendregt J.J., Willekens F., Mackenbach J.P., Al Mamun A., Bonneux L. (2003). Obesity in adulthood and its consequences for life expectancy: A life-table analysis. Ann. Intern. Med..

[29] Peeters A., Bonneux L., Nusselder W.J., de Laet C., Barendregt J.J. (2004). Adult obesity and the burden of disability throughout life. Obes. Res..

[30] Keating S.E., Coombes J.S., Stowasser M., Bailey T.G. (2020). The Role of Exercise in Patients with Obesity and Hypertension. Curr. Hypertens. Rep..

[31] Bray G.A., Kim K.K., Wilding J.P.H., World Obesity Federation (2017). Obesity: A chronic relapsing progressive disease process. A position statement of the World Obesity Federation. Obes. Rev..

[32] Warburton D.E., Nicol C.W., Bredin S.S. (2006). Health benefits of physical activity: The evidence. CMAJ.

[33] Ekelund U., Tarp J., Fagerland M.W., Johannessen J.S., Hansen B.H., Jefferis B.J., Whincup P.H., Diaz K.M., Hooker S., Howard V.J. (2020). Joint associations of accelero-meter measured physical activity and sedentary time with all-cause mortality: A harmonised meta-analysis in more than 44 000 middle-aged and older individuals. Br. J. Sports Med..

[34] Oppert J.M., Bellicha A., van Baak M.A., Battista F., Beaulieu K., Blundell J.E., Carraça E.V., Encantado J., Ermolao A., Pramono A. (2021). Exercise training in the management of overweight and obesity in adults: Synthesis of the evidence and recommendations from the European Association for the Stuy of Obesity Physical Activity Working Group. Obes. Rev..

